# Genetically Engineered Brain Organoids Recapitulate Spatial and Developmental States of Glioblastoma Progression

**DOI:** 10.1002/advs.202410110

**Published:** 2025-01-21

**Authors:** Matthew Ishahak, Rowland H. Han, Devi Annamalai, Timothy Woodiwiss, Colin McCornack, Ryan T. Cleary, Patrick A. DeSouza, Xuan Qu, Sonika Dahiya, Albert H. Kim, Jeffrey R. Millman

**Affiliations:** ^1^ Division of Endocrinology Metabolism and Lipid Research Washington University School of Medicine 660 South Euclid Avenue, Campus Box 8127 St. Louis MO 63110 USA; ^2^ Department of Genetics Washington University School of Medicine 4515 McKinley Ave. St. Louis MO 63110 USA; ^3^ Department of Neurological Surgery University of Iowa Healthcare 1800 John Pappajohn Pavilion Iowa City IA 52242 USA; ^4^ Division of Neuropathology Washington University School of Medicine 660 South Euclid Avenue, Campus Box 8118 St. Louis MO 63110 USA; ^5^ Taylor Family Department of Neurosurgery Washington University School of Medicine 660 South Euclid Avenue, Campus Box 8057 St. Louis MO 63110 USA; ^6^ The Brain Tumor Center at Siteman Cancer Center 4921 Parkview Place St. Louis MO 63110 USA; ^7^ Department of Biomedical Engineering Washington University 1 Brookings Drive, Campus Box 1097 St. Louis MO 63130 USA

**Keywords:** brain organoids, genetic engineering, glioblastoma, single‐cell RNA sequencing, spatial sequencing

## Abstract

Glioblastoma (GBM) is an aggressive form of brain cancer that is highly resistant to therapy due to significant intra‐tumoral heterogeneity. The lack of robust in vitro models to study early tumor progression has hindered the development of effective therapies. Here, this study develops engineered GBM organoids (eGBOs) harboring GBM subtype‐specific oncogenic mutations to investigate the underlying transcriptional regulation of tumor progression. Single‐cell and spatial transcriptomic analyses revealed that these mutations disrupt normal neurodevelopment gene regulatory networks resulting in changes in cellular composition and spatial organization. Upon xenotransplantation into immunodeficient mice, eGBOs form tumors that recapitulate the transcriptional and spatial landscape of human GBM samples. Integrative single‐cell trajectory analysis of both eGBO‐derived tumor cells and patient GBM samples reveal the dynamic gene expression changes in developmental cell states underlying tumor progression. This analysis of eGBOs provides an important validation of engineered cancer organoid models and demonstrates their utility as a model of GBM tumorigenesis for future preclinical development of therapeutics.

## Graphical abstract

This study develops engineered glioblastoma organoids to investigate the role of specific mutations in tumor progression. The analytic framework spans single‐cell analysis, spatial transcriptomics, single‐cell trajectory analysis, orthotopic implantation, clinically oriented imaging, and histopathological analysis. The work provides an important proof of concept that engineered tumor organoids can model glioblastoma progression.

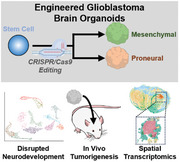

## 1 Introduction

Mapping and understanding the genetic drivers of tumorigenesis and tumor progression have been fundamental goals in the study of cancer. The Cancer Genome Atlas (TCGA), which was started in 2006, has performed molecular characterization of over 20000 primary tumors from thirty‐three cancer types.^[^ ^1^ ^]^ Glioblastoma (GBM), the first cancer studied by TCGA,^[^ ^2^ ^]^ is the most common primary malignant brain tumor in adults, accounting for over 14% of all brain and other central nervous system tumors in the United States.^[^ ^3^ ^]^ Patients with GBM have a poor prognosis, with a median survival of less than two years after diagnosis despite rigorous therapy.^[^ ^4^ ^]^ To date, TCGA has conducted comprehensive multidimensional analysis of over six hundred GBM tumors, resulting in the classification of GBM into Proneural, Neural, Classical, and Mesenchymal subtypes.^[^ ^5^ ^]^ Despite thorough characterization of patient samples, very few new therapeutic options for GBM have been developed in last the decade,^[^ ^6^ ^]^ which highlights the need for improved preclinical models.

Currently, the most widely used preclinical models for brain tumors include genetically modified mice, syngeneic mouse models, and patient‐derived cancer spheroids.^[^ ^7^ ^]^ Genetic modification of tumor suppressor genes and introduction of oncogenic perturbations in mouse models result in the formation of GBM‐like tumors. However, species‐specific differences in brain morphology and physiology combined with the differences in tumor growth patterns and antigen expression limit the human relevance of mouse models. While patient‐derived 3D tumor spheroids can recreate the cellular heterogeneity of GBM, these models are often derived from late‐stage tumors which hinder investigation of early tumorigenesis processes due to the high mutational burden that occurs over time.^[^ ^8^ ^]^

Recently, human pluripotent stem cell‐derived brain organoids have emerged as a powerful in vitro system to model human neural development and diseases.^[^ ^9^ ^]^ Brain organoids are of particular interest for modeling cerebral tumors, since it has been observed that tumor cell types are more closely associated with developmental‐like signatures, rather than adult cell populations^[^ ^10^ ^]^ and that neural development transcriptional programs govern invasion.^[^ ^11^ ^]^ Previously, CRISPR‐Cas9‐based methods have been utilized to introduce oncogenic mutations into developing organoids resulting in neoplastic cerebral organoids.^[^ ^7^, ^12^ ^]^ While these model systems exhibit many features of cancer, it remains unclear how well they recapitulate human GBM tumorigenesis and if the resulting tumors are comparable to advanced GBM patient tumors. As a result, there is a need for further development and characterization of engineered organoid models of GBM.

Here, we generated engineered GBM brain organoids (eGBOs) from genetically modified stem cells harboring mutations of both the Proneural and Mesenchymal subtypes of GBM. Using single‐cell RNA sequencing (scRNAseq) and spatial transcriptomic analyses, we demonstrate that GBM‐associated mutations alter neural development in brain organoids. Further, we find that transplantation of cells from eGBOs into mice results in tumors that recapitulate subtype‐specific characteristics of human GBM tumors. Finally, we identify dynamic gene expression changes in developmental cell states underlying tumor progression using integrated single‐cell transcriptomic data from eGBOs and patient GBM tumors. These eGBOs are an effective model of GBM tumorigenesis and, combined with the robust framework for computational analysis we describe, provide an important validation of engineered cancer organoid models for future development of cancer therapies.

## 2 Results

### 2.1 Generation of eGBOs from Stem Cells Harboring GBM‐Associated Mutations

**Figure 1:**
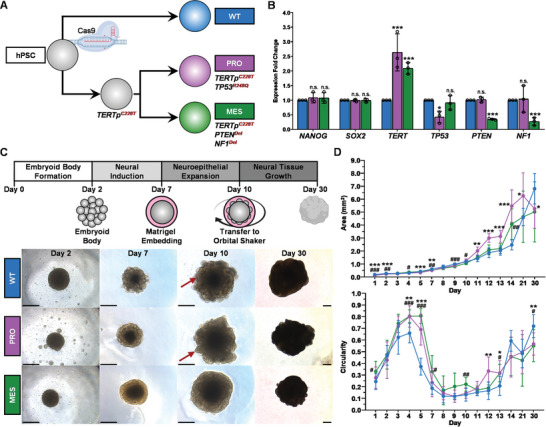
Generation of eGBOs from hESCs harboring GBM‐associated mutations. A) Genetic engineering strategy to generate eGSCs by introducing GBM‐associated mutations into hPSCs via sequential CRISPR‐Cas9 gene editing. B) qPCR analysis of genetically engineered hPSCs indicating unchanged expression of pluripotency markers and altered expression of genes targeted by CRISPR editing. Statistical analysis by parametric unpaired t‐test versus WT condition with Holm‐Sidak correction for multiple comparisons (n = 3 biological replicates). Error bars = standard deviation. *P < 0.05; **P < 0.01; ***P < 0.0001 C) Schematic of brain organoid differentiation protocol and brightfield images during cerebral organoid differentiation. Red arrowheads indicate neuroepithelial growth, which is slightly reduced in PRO organoids, and greatly reduced in MES organoids. Scale bars = 500 µm. D) Surface area (top) and circularity (bottom) measurements during organoid differentiation (n = 10 individual organoids). Statistical analysis by two‐way ANOVA using Geisser‐Greenhouse correction and Tukey correction for multiple comparison (* indicates WT vs PRO comparisons and # indicates WT vs MES comparisons). Error bars = standard deviation. *P < 0.05; **P < 0.01; ***P < 0.0001.

Prior characterization of the transcriptomes from human GBM tumors identified genetic subtypes of GBM with specific mutations associated with each subtype.^[^ ^5^ ^]^ Therefore, we first sought to genetically engineer stem cells to mimic the heterogeneous genetic landscape of GBM. Through serial introduction of GBM‐associated mutations in healthy human pluripotent stem cells (hPSCs) using CRISPR‐Cas9, we developed two engineered GBM stem cell (eGSC) lines harboring representative mutations of the Proneural (PRO) and Mesenchymal (MES) GBM subtypes (**Figure** **1**A; Figure S1A, Supporting Information). First, hPSCs were transfected with a CRISPR‐Cas9 ribonucleoprotein complex to introduce the machinery necessary for precise genetic modification. We then introduced a C228T mutation in the telomerase reverse transcriptase (*TERT*) promoter region to create a genetic background that promotes rapid tumor progression through reactivation of the *TERT* gene, a characteristic of GBM progression due to binding of E‐twenty‐six (ETS) transcription factors that may be coordinated by a heterotetrameric GABP transcription factor complex or non‐canonical NF‐κB signaling.^[^ ^8^, ^13^ ^]^

The p53 pathway is dysregulated in ≈85% of all GBM tumors^[^ ^14^ ^]^ and has been implicated in invasion, migration, proliferation, evasion of apoptosis, and cancer cell stemness.^[^ ^15^ ^]^ Despite being the most frequently gene mutated in GBM, *TP53* mutations are mostly commonly observed in the PRO subtype of GBM.^[^ ^5^ ^]^ Therefore, we introduced a R248Q hotspot mutation in the *TP53* gene to recapitulate the genetic profile of the PRO GBM subtype.

Decreased expression of *NF1* is characteristic of the MES subtype of GBM,^[^ ^5^, ^16^ ^]^ caused by loss of the chromosome region containing the *NF1* gene. Additionally, the *PTEN* gene has been identified as a tumor suppressor that is recurrently mutated in GBM,^[^ ^17^ ^]^ with deletion or mutation leading to dysregulation of the PI3K pathway.^[^ ^18^ ^]^ Concomitant mutation of *NF1* and *PTEN* is found almost exclusively within MES subtype tumors,^[^ ^5^ ^]^ therefore small deletions were introduced to the *NF1* and *PTEN* genes to recreate the genetic profile of the MES GBM subtype.

After introducing GBM‐associated mutations, we validated that eGSCs maintained normal stem cell morphology and pluripotency marker expression. Quantitative PCR analysis confirmed that CRISPR genetic modifications significantly altered target gene expression, without altering expression of pluripotency markers (Figure 1B). A twofold increase in *TERT* expression was observed in both the PRO and MES eGSCs compared to wildtype (WT) hPSCs. Additionally, expression of *TP53* was significantly decreased in the PRO eGSCs, while both *NF1* and *PTEN* expression were significantly decreased in the MES eGSCs. Further, there was no significant difference in the expression of the stem cell marker genes, *NANOG* and *SOX2*. Both PRO and MES eGSCs also maintained characteristic stem cell morphology and expression of NANOG protein (Figure S1B, Supporting Information). Next‐generation sequencing (NGS) further confirmed highly efficient modifications in the *TP53*, *PTEN*, and *NF1* genes, and the *TERT* promoter region (Figure S1C, Supporting Information). These results demonstrate that GBM subtype‐specific mutations can be introduced into hPSCs without altering stemness.

Previous reports have proposed that GBM hijacks normal neural development mechanisms to drive tumor growth.^[^ ^11^ ^]^ Therefore, we differentiated eGSCs into brain organoids, following an established protocol with minor modifications, to investigate how GBM‐associated mutations may alter neural development (Figure 1C; Table S1, Supporting Information).^[^ ^9^, ^19^ ^]^ Visual assessment of developing organoids by bright‐field imaging revealed slight morphological differences between WT organoids and eGBOs (Figure 1C). Notably, MES eGBOs lacked neuroepithelial growth on day 10. This observation is consistent with previous reports that disruption of the *PTEN* gene leads to delayed differentiation and altered surface folding in cerebral organoids.^[^ ^20^ ^]^ We also performed assessment of surface area and circularity to quantitatively assess developing organoids (Figure 1D). During the initial days of differentiation, WT organoids and eGBOs were approximately the same size. Around day 6, however, developing eGBOs were significantly smaller than WT organoids. This corresponded with eGBOs maintaining a highly circular morphology during the transition from embryoid body formation to the neural induction stage and, while developing PRO eGBOs were not significantly different from developing WT organoids on day 10, developing MES eGBOs maintained this circular morphology. Despite these differences in growth dynamics, our data demonstrates that brain organoids were successfully generated from both WT hPSCs and eGSCs harboring GBM subtype‐specific mutations.

### 2.2 Transcriptional landscape of Neural Development is Altered in eGBOs

Differentiation of hPSCs into brain organoids results in multiple cell types representing the developing cerebral cortex, ventral telencephalon, retina, and other regions of neural development. Therefore, we performed scRNAseq to characterize the cellular composition and transcriptional profile of WT organoids and eGBOs. We implemented a cell hashing approach to sequence all three genotypes in a single, multiplexed batch, thereby reducing technical variability (Figure S2A, Supporting Information). A total of 9310 single cells were sequenced at an average read depth of 58696 reads per cell and a median of 2857 genes per cell. Cells with less than 200 genes detected and genes detected in less than three cells were excluded from downstream analysis. We achieved a hashing efficiency of ≈77% with 7155 cells identified by a single hashtag, while 1062 cells and 1085 were identified as negative and doublets, respectively. After filtering out negative and doublet cells, expression of a single hashtag enabled clear delineation of cells from WT, PRO, and MES organoids (Figure S2B, Supporting Information). Further quality control filtering to remove low‐quality cells based on the percentage of mitochondrial genes resulted in 5073 high‐quality cells for further analysis. Pseudobulk analysis of GBM‐associated genes targeted for mutation further validated our hashing methodology, with increased *TERT* expression observed in eGBOs compared to WT organoids and reduced expression of *TP53* in PRO eGBOs and both *PTEN* and *NF1* in MES eGBOs (Figure S2C, Supporting Information).

**Figure 2:**
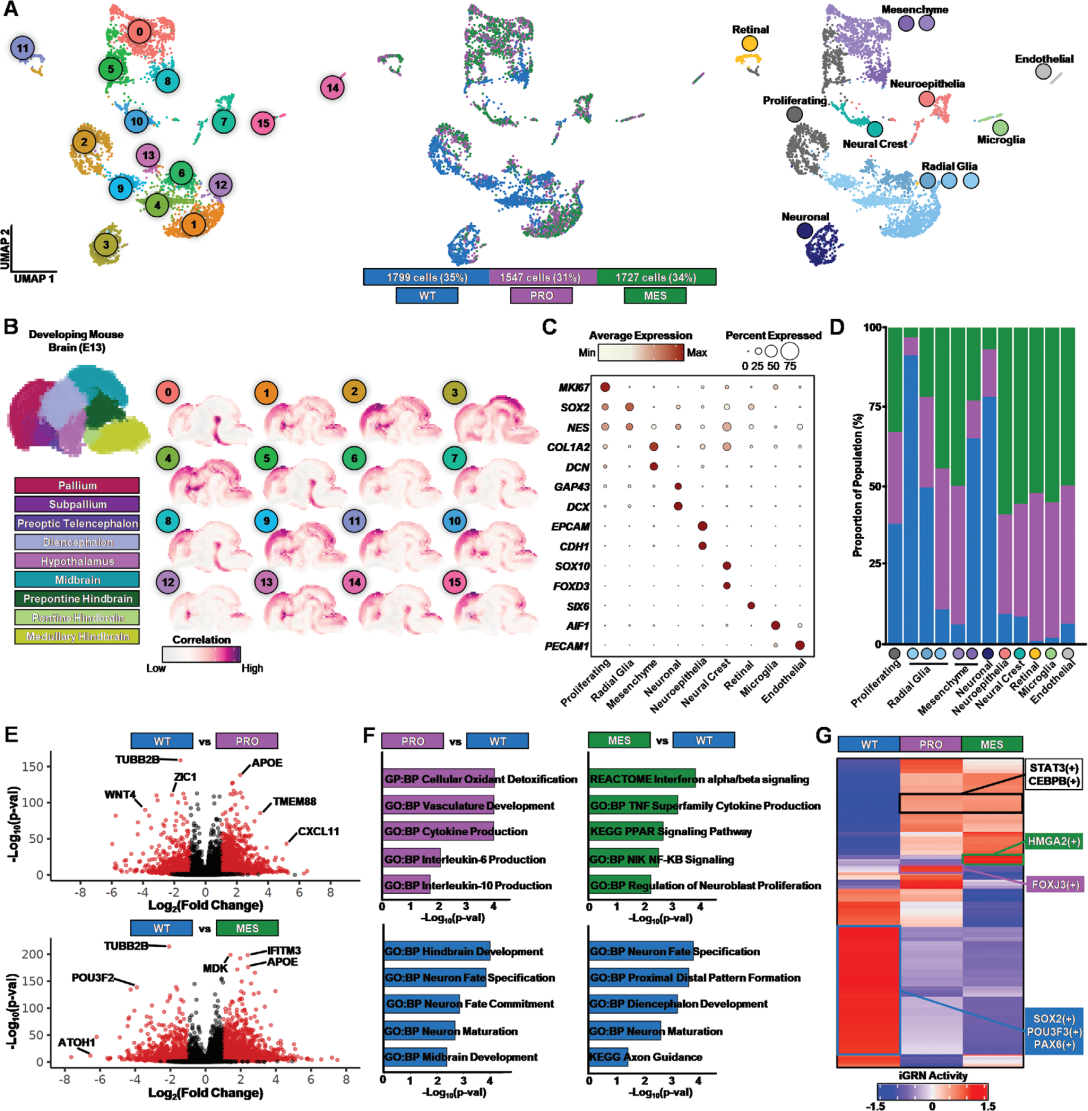
GBM mutations alter neural development gene regulatory networks in eGBOs. A) UMAPs showing Louvain clusters (left), sample identification (middle), and cell types (right) in brain organoids sequenced after 1 month of in vitro differentiation (5073 total cells from WT, PRO and MES organoids). B) Spatial location inference of Louvain clusters identified in brain organoids. Reference regions provided by the Developing Mouse Brain database of Allen Brain Atlas (left) and correlation patterns of average regional marker gene expression for each Louvain cluster (right). C) Dot plot indicating expression of marker genes that define cell types observed in organoids. D) Stacked bar plot showing the proportion of cells from WT organoids and eGBOs for each cell type. E) Volcano plots for indicated pairwise comparisons from scRNAseq data. Red dots represent significantly differentially expressed transcripts (P<0.05) with Fold Change≥1. F) GSEA pathways from differentially expressed transcripts for indicated pairwise comparisons. G) Heatmap indicating scaled activity of iGRNs in WT, PRO, and MES organoids.

Unsupervised, hierarchical clustering and embedding into a 2D space with uniform manifold approximation and projection (UMAP) enabled annotation of cell types and revealed differential clustering of cells from WT organoids and eGBOs (**Figure** **2**A). We identified sixteen distinct cell populations using the Louvain clustering algorithm. The gene expression profiles of these clusters were highly correlated to the pallium and midbrain regions of the developing brain, regardless of the cluster consisting of cells from WT organoids or eGBOs (Figure 2B; Figure S2D, Supporting Information). Cell types within WT organoids and eGBOs were annotated by a combination of supervised assessment of marker gene expression and unsupervised comparison to reference datasets (Figure 2C; Figure S2E,F, Supporting Information). We observed clearly defined cell populations of normal brain organoid development, including radial glial cells (expressing *SOX2* and *NES*), neuronal cells (expressing *GAP43* and *DCX*), and mesenchymal cells (expressing *COL1A2* and *DCN*). We also identified off‐target populations commonly found in brain organoids, including neural crest cells (expressing *SOX10* and *FOXD3*), retinal progenitors (expressing *SIX6*), microglia (expressing *C1QC*), and endothelial cells (expressing *PECAM1*). Notably, these off‐target populations primarily consisted of cells derived from both PRO and MES eGBOs, while the majority of the neuronal population consisted of cells derived from WT organoids (Figure 2D).

Next, we identified differentially expressed genes between WT organoids and eGBOs (Table S2, Supporting Information). We observed common neural development genes upregulated in WT organoids compared to eGBOs, (Figure 2E). Specifically, *TUBB2B*, which encodes *β*‐tubulin found in neuronal axons,^[^ ^21^ ^]^ *ZIC1*, which regulates forebrain development,^[^ ^22^ ^]^ *WNT4*, which regulates axonal growth,^[^ ^23^ ^]^ *POU3F2*, which is involved in cortical development,^[^ ^24^ ^]^ and *ATOH2*, which governs development of various neuronal subtypes,^[^ ^25^ ^]^ were all significantly upregulated in WT organoids compared to eGBOs. In contrast, we observed genes implicated in GBM progression significantly upregulated in eGBOs compared to WT organoids (Figure 2E). In particular, *APOE*,^[^ ^26^ ^]^ *TMEM88*,^[^ ^27^ ^]^ and *CXCL11* ^[^ ^28^ ^]^ were significantly upregulated in PRO eGBOs compared to WT organoids. the significantly upregulated genes in MES eGBOs compared to WT organoids included *APOE*,^[^ ^26^ ^]^ *IFITM3*,^[^ ^29^ ^]^ and *MDK*.^[^ ^30^ ^]^ We also performed gene set enrichment analysis (GSEA) on differentially expressed genes with a Log_2_(Fold Change) > 1.0. This analysis revealed that genes upregulated in WT organoids were significantly enriched in gene sets across multiple lists from the Molecular Signatures Database (MSigDB) associated with normal neural development, while genes upregulated in eGBOs were significantly enriched in gene sets associated with cancer pathways (Figure 2F).

To better understand the underlying networks driving the observed transcriptional differences between WT organoids and eGBOs, we performed inferred gene regulatory networks (iGRN) analysis (Figure 2G; Figure S3, Supporting Information). This method quantifies transcription factor (TF) activity by constructing regulons, which consist of a transcription factor and its downstream target genes based on co‐expression inferencing.^[^ ^31^ ^]^ We identified 411 regulons and observed distinctive patterns in regulon activity between WT organoids and eGBOs (Table S3, Supporting Information). UMAP clustering based on iGRN activity further delineated the transcriptional differences between WT organoids and eGBOs (Figure S3A, Supporting Information). Notably, highly active regulons specific to WT organoids include TFs associated with brain patterning in developing organoids,^[^ ^32^ ^]^ including SOX2(+), POU3F3(+), and PAX6(+) (Figure 2G; Figure S3B, Supporting Information), while TFs shown to be synergistic master regulators that promote tumorigenesis and invasion in gliomas,^[^ ^33^ ^]^ such as STAT3(+) and C/EBP*β*(+), were among the highly active regulons specific to both the PRO and MES eGBOs (Figure 2G; Figure S3B, Supporting Information). Interestingly, the most variable regulon within this dataset was PITX2(+), which is required for normal neuron development^[^ ^34^ ^]^ and associated with poor survival in GBM patients.^[^ ^35^ ^]^ Further, high regulon activity for TFs that have been implicated in cancer stem cells was observed in both eGBOs. MES eGBOs had high activity of the HMGA2(+) regulon, which promotes GBM stemness, invasion and in vivo tumor formation^[^ ^36^ ^]^ (Figure 2G), while PRO eGBOs had high activity of the FOXJ3(+) regulon, which is a neuroectodermal TF recently identified as a GBM‐driving gene.^[^ ^37^ ^]^ Together, these results demonstrate that underlying differences in gene regulatory networks alter the neural development of eGBOs and promote an oncogenic transcriptional landscape.

### 2.3 Cellular Architecture is Disrupted in Developing eGBOs

To understand how the altered transcriptional landscape of eGBOs affects spatial organization in brain organoids, we performed in situ sequencing (ISS) using the Xenium platform (10x Genomics) to generate high‐resolution spatial gene expression maps using a targeted panel of 266 genes (Table S4, Supporting Information). This enabled spatial localization of genes critical for neural development^[^ ^38^ ^]^ and GBM progression,^[^ ^39^ ^]^ such as *NES, PAX6, MEIS2, SOX11*, *SOX2*, *SOX9*, *EGFR* and *CDH1* (Figure S4A, Supporting Information).

**Figure 3:**
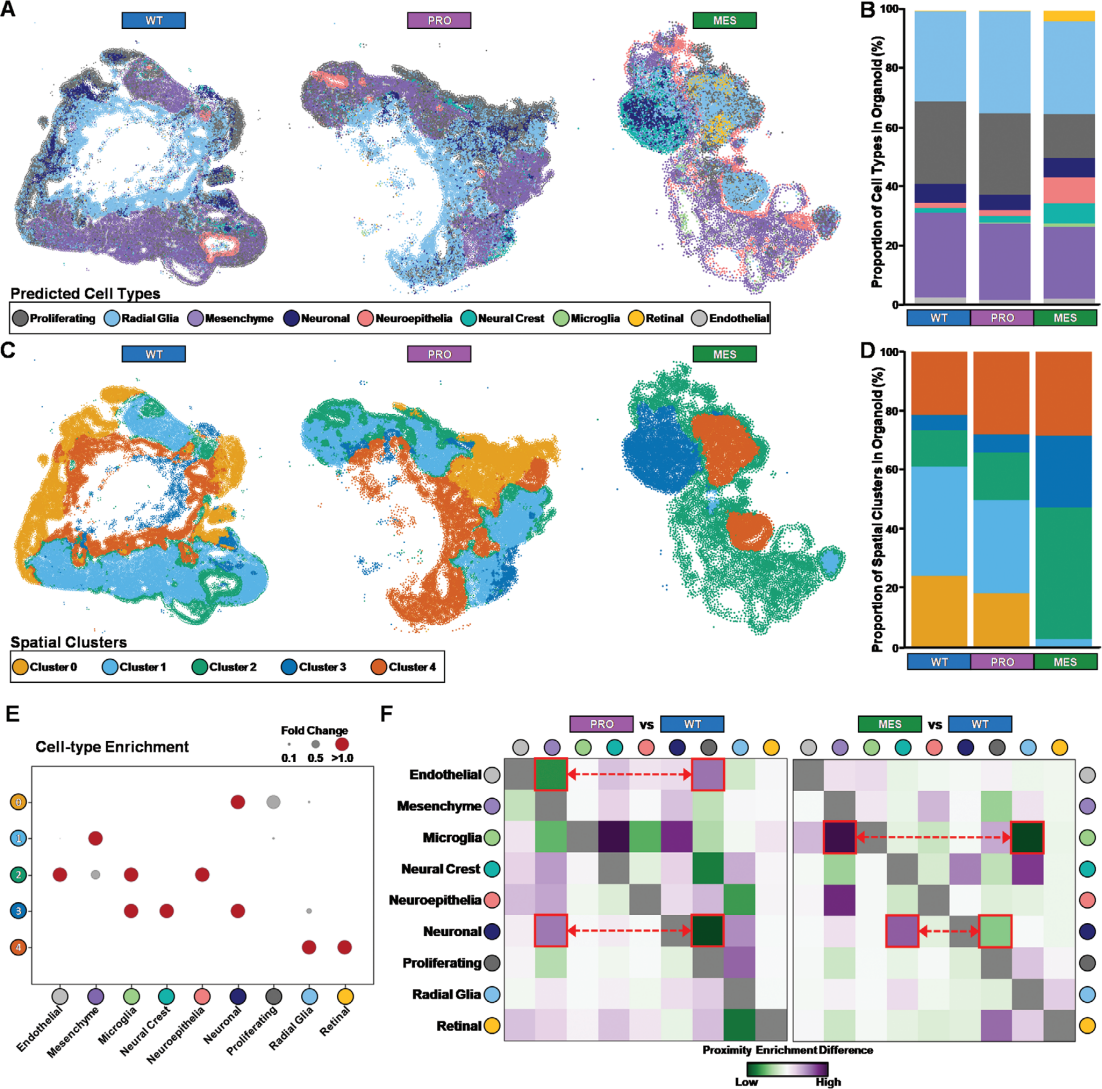
Spatial sequencing analysis of eGBOs. A) Spatial distribution of cell types predicted from scRNAseq data in WT, PRO, and MES organoids. B) Stacked bar plot showing the proportion of cell types in WT, PRO, and MES organoids. C) Spatial distribution of clusters determined by cluster stability analysis. D) Stacked bar plot showing the proportion of spatial clusters in WT, PRO, and MES organoids. E) Cell‐type enrichment in each spatial cluster. F) Differential neighborhood enrichment heatmap between spatial distribution of major cell types in WT and eGBOs. Examples of differentially enriched neighborhoods are highlighted in red.

While ISS provides high spatial resolution of gene expression, the limited panel of target genes creates challenges for robust downstream analyses, such as cell type annotation. Therefore, we applied the anchor‐based integration workflow, implemented in Seurat,^[^ ^40^ ^]^ to perform probabilistic transfer of cell type annotations from scRNAseq data generated from batch‐matched organoids. Visualization of cell type annotations in WT organoids revealed an expected morphological arrangement, with radial glia cells located toward the center of the developing organoid, surrounded by proliferating and neuronal‐like cells (**Figure** **3**A). In both PRO and MES eGBOs this architecture is disrupted, with less clear delineation of developing neuronal regions around radial glial populations (Figure 3A). Interestingly, the proportions of radial glial, mesenchyme, and neuronal cells were similar in WT organoids and eGBOs (Figure 3B). However, a greater proportion of off‐target cell types was observed in eGBOs, especially the MES eGBO, which had much larger neuroepithelial, neural crest, retinal, and microglia populations.

Next, we performed spatial clustering to define regions based on the transcriptional profile of both individual cells and cells in the surrounding neighborhood.^[^ ^41^ ^]^ We identified five spatially defined clusters, based on cluster stability analysis, across both WT organoids and eGBOs (Figures 3C and S4B, Supporting Information). Interestingly, WT organoids and PRO eGBOs consisted of similar proportions of each spatial cluster (Figure 3C,D). In contrast, MES eGBOs consisted of larger proportions of Cluster 2 and Cluster 3 and decreased proportions of Cluster 0 and Cluster 1 (Figure 3C,D). To characterize the composition of spatially defined clusters, we calculated cell‐type enrichments within each spatial cluster. Expectedly, each spatial cluster was associated with a unique combination of cell types (Figure 3E). Cluster 0 clearly marked a developing neuronal region, indicated by enrichment of neuronal and proliferating cell types. Interestingly, this cellular niche was absent from MES eGBOs and slightly reduced in PRO eGBOs. Cluster 1, marking primarily mesenchymal cells, was also much smaller in MES eGBOs and slightly reduced in PRO eGBOs. Multiple cell types, mostly off‐target cell populations, were enriched in Cluster 2 and Cluster 3 and comprised a larger proportion of the eGBOs than they did in WT organoids. Additionally, Cluster 4, which primarily consisted of radial glial cells, was slightly larger in both eGBOs compared to WT organoids.

**Figure 4:**
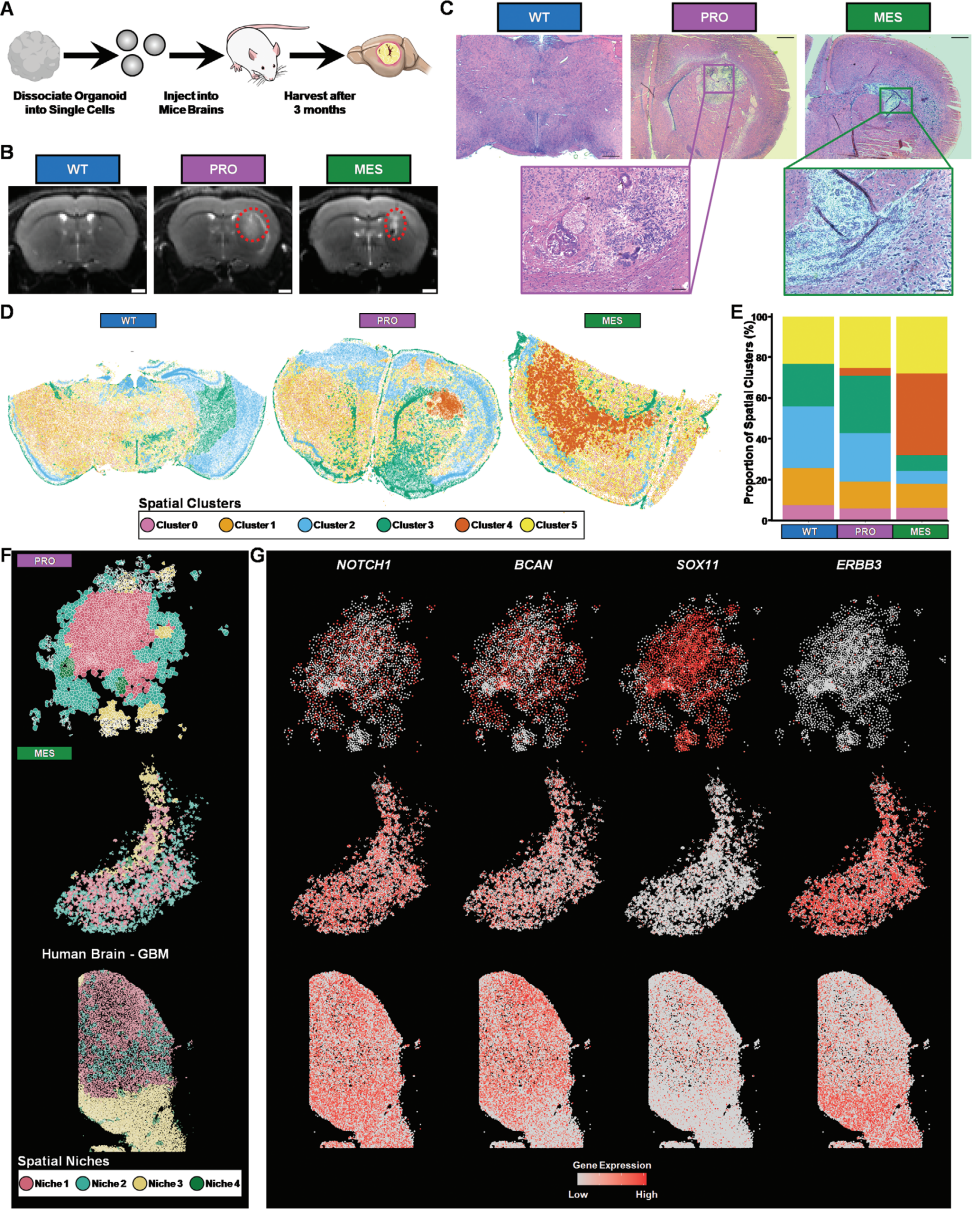
Spatially resolved transcriptomics analysis of eGBO‐derived tumors. A) Experimental plan to assess tumorigenicity of eGBOs. B) Representative T2‐post contrast MRIs of mouse brains 3 months post‐transplantation of dissociated eGBO cells. Red, dotted circles indicate contrast‐enhancing lesions; Scale bars = 1 mm C) H&E staining of brain sections demonstrating glioma‐like growths in mice that received cells from PRO and MES eGBOs (Scale bars = 500 µm). Insets highlight characteristic histopathology of malignant tumors (Scale bars = 100 µm). D) Spatial distribution of clusters determined by cluster stability analysis in recovered mouse brains. E) Stacked bar plot showing the proportion of spatial clusters identified in recovered mouse brains. F) Spatially defined niches within tumor regions identified in mice that received cells from either PRO or MES eGBOs and a patient tumor sample. G) Spatial distribution of genes associated with GBM development and migration in eGBO‐derived tumors and a patient tumor sample.

To determine changes in the spatial proximity of the different cell populations between WT organoids and eGBOs, we performed neighborhood enrichment analysis (Figure S4C, Supporting Information). We also, compared the differences in neighborhood enrichment between eGBOs and WT organoids across cell populations (Figure 3F). In PRO eGBOs, neuronal cells decreased interaction with proliferating cells, in favor of interactions with mesenchymal cell types, compared to WT organoids. Additionally, there was an increased interaction of endothelial cells with proliferating cells, suggesting pro‐angiogenic signaling, compared to WT organoids. In MES eGBOs, neuronal cells decreased interaction with proliferating cells and instead interacted more closely with neural crest cells, compared to WT organoids. Also, microglia cells strongly shift away from radial glial, favoring interaction with mesenchymal cells, in MES eGBOs compared to WT organoids. Together, these data demonstrate that the cellular architecture of developing brain organoids is altered in eGBOs.

### 2.4 eGBOs form Glioma‐like Tumors In Vivo

To assess if the observed transcriptional and architectural differences of eGBOs were indicative of tumor initiating cells, we tested the oncogenic potential of these cells in vivo. Approximately 2.6 × 10^5^ cells were stereotactically injected into the base of the right forebrain of athymic mice (**Figure** **4**A). Magnetic resonance imaging (MRI) was performed to track tumor formation and growth (Figure 4B; Figure S5A, Supporting Information). Clear contrast‐enhancing lesions were observed in all mice that were injected with PRO eGBO cells and 80% of mice that were injected with MES eGBO cells at 3 months post‐transplantation (Figure S5B, Supporting Information). On average, the tumors observed by MRI in mice that received PRO eGBO cells were larger (1.61 ± 0.96 mm^2^) than those observed in mice that received MES eGBO cells (0.69 ± 1.07 mm^2^). Hematoxylin & Eosin (H&E) staining in harvested brains showed malignant tumor in both PRO and MES eGBO conditions (Figure S5C, Supporting Information). Additional special stains indicated that both the PRO and MES tumors were positive for GFAP and VIM (Figure S5D, Supporting Information). The PRO eGBO‐derived tumor appeared to contain divergent differentiation along multiple lineages with the presence of pigmented epithelium and dominance of glial elements. Interestingly, the MES eGBO‐derived tumors were less apparent on MRI scans, which was further confirmed by histology, but these tumors appeared far more infiltrative beyond their epicenters; they also showed conspicuous spindled/mesenchymal phenotype in the epicenter. We utilized a deep learning model (https://gbm360.stanford.edu/)^[^ ^42^ ^]^ to characterize cellular heterogeneity and predict prognosis based on the histology of observed growths (Figure S6A, Supporting Information). In both PRO and MES eGBO conditions, the machine learning algorithm identified regions of tumor cells corresponding to the observed glioma‐like growths. The PRO tumor consisted primarily of neural progenitor‐like (NPC‐like) GBM cells, while the MES tumor consisted of a mix of NPC‐like, oligodendrocyte‐progenitor‐like (OPC‐like), astrocyte‐like (AC‐like), and mesenchymal‐like (MES‐like) cells. Further, the model assigned a moderate to high aggressiveness score to the cells in these regions.

We performed ISS on recovered mouse brains using the 10x Xenium Human Brain panel to characterize the spatial transcriptional profile of observed growths. There are 34 tumor‐associated genes in the 10x Xenium Human Brain panel. The localization of these transcripts identified tumor regions in both eGBO conditions (Figure S6B, Supporting Information). To further characterize spatial patterning of gene expression, we calculated the Moran's I global spatial auto‐correlation statistics for all genes (Table S5, Supporting Information). Comparing genes with a Moran's I statistic greater than 0.1 revealed that eight genes were spatially autocorrelated in both eGBO conditions (Figure S6C, Supporting Information). Among these shared genes were the most highly spatially autocorrelated genes, *NNAT* and *IGFBP3*, in the PRO and MES conditions, respectively, which have both been associated with GBM progression and reduced patient survival.^[^ ^43^ ^]^

Spatial clustering revealed six regions across brain sections recovered from mice injected with cells derived from WT, PRO, or MES organoids (Figure 4D; Figure S6D, Supporting Information). These clusters represented various brain regions including cortical layers, indicated by expression of *CUX2*, *RORB*, and *LAMP5*, and the dorsal thalamus, marked by expression of *NTNG1* ^[^ ^44^ ^]^ (Figure S6E, Supporting Information). Notably, Cluster 4, which was present only in mice injected with cells from eGBOs, clearly marked regions aligning with glioma‐like growths observed in MRI scans and H&E staining (Figure 4D,E).

To further characterize the spatial transcriptomic profile of tumors generated by eGBOs, we computed spatial niches within the spatial cluster corresponding to glioma‐like growths, revealing distinct spatial organization in PRO and MES eGBO‐derived tumors (Figure 4F). Recent integrative spatial analysis demonstrated that human GBM consists of both structured and disorganized regions.^[^ ^45^ ^]^ Based on the distribution of spatial niches in eGBO‐derived tumors, we find that the PRO eGBO‐derived tumor has a more structured organization, while the MES eGBO‐derived tumor was more disorganized. Both PRO and MES eGBO‐derived tumors had broad expression of *NOTCH1*, which is commonly upregulated in glioma tumor‐initiating cells,^[^ ^46^ ^]^ and *BCAN*, which encodes an extracellular matrix protein found in human gliomas^[^ ^47^ ^]^ (Figure 4G). However, expression of *SOX11*, a TF crucial for brain development that is also overexpressed in GBM,^[^ ^48^ ^]^ appeared to be localized to the core of the PRO eGBO‐derived tumor, while the MES eGBO‐derived tumor broadly expressed *ERBB3*, a subtype‐specific GBM marker.^[^ ^49^ ^]^ Notably, we observed a similar spatial expression pattern for all of these markers in a patient GBM sample. Together, these data demonstrate that eGBOs can generate tumors that demonstrate subtype‐specific characteristics of human GBM tumors.

### 2.5 eGBO‐Derived Tumors Recapitulate Cell States Found in Human GBM

To characterize the transcriptional profile of the eGBO‐derived tumors more thoroughly, we performed scRNAseq on dissociated cells isolated using a brain tumor dissociation kit. Substantially fewer single cells were recovered from mice that received cells from WT organoids compared to mice that received cells from eGBOs. Additionally, the mean genes per cell from cells recovered from mice treated with WT organoid cells was greatly reduced compared to cells recovered from mice treated with eGBOs. These data suggest mouse cells were most likely recovered from the WT organoid condition, due to the low mapping of human genes, and further suggest that WT organoids do not form glioma‐like growths in vivo. Following quality control filtering, 476 high‐quality cells were recovered from transplanted WT organoids while 10103 high‐quality cells and 12452 high‐quality cells were recovered from transplanted PRO and MES eGBOs, respectively, for further analysis.

**Figure 5:**
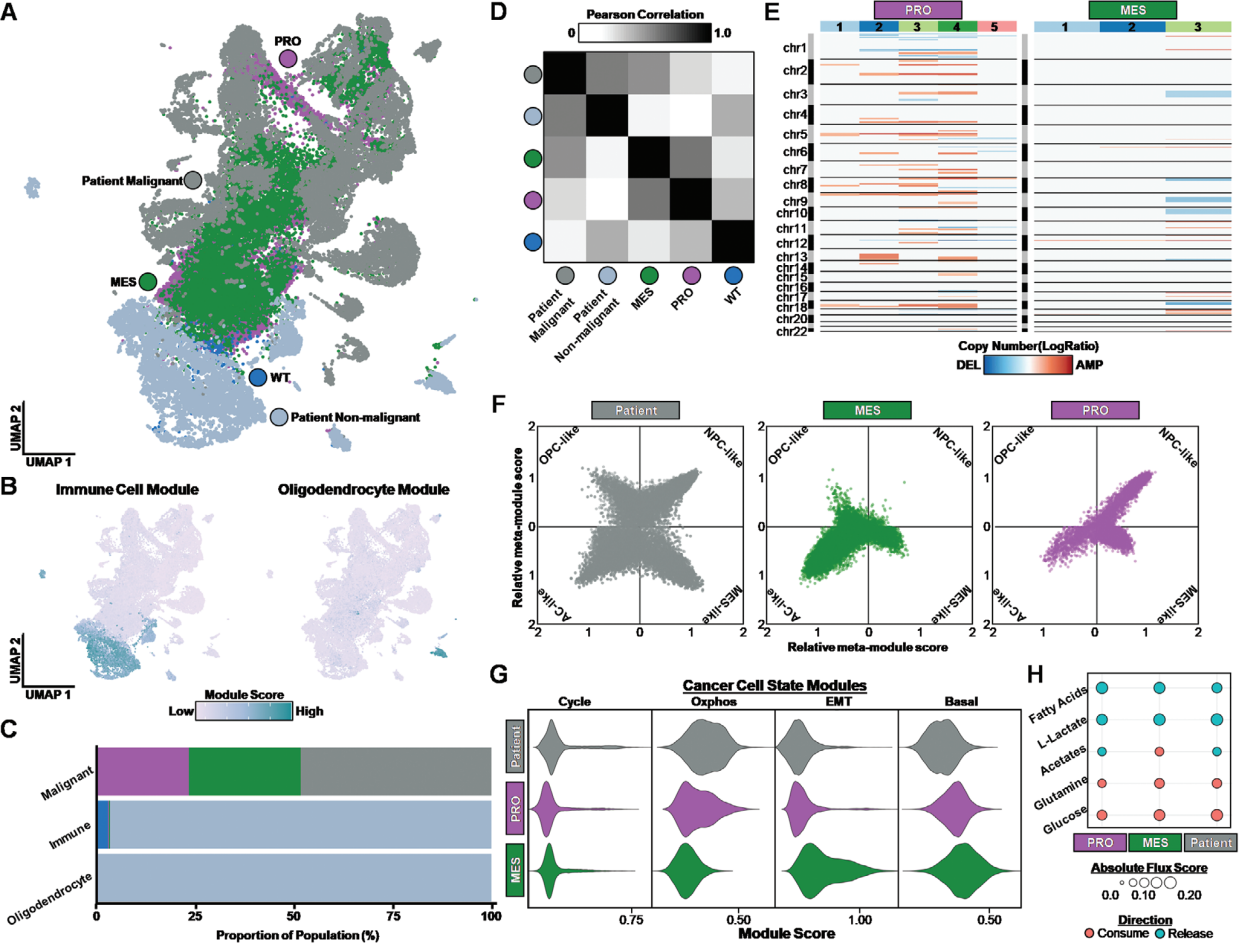
Integrative transcriptional profiling of eGBO‐derived tumors and patient GBM samples. A) UMAP of integrated recovered WT organoid cells, eGBO‐derived tumors, and GBM patient samples. B) Feature plots indicating module scores for non‐malignant cell types present in GBM samples. C) Stacked bar plot showing the distribution of each sample across malignant and non‐malignant cell types. D) Heatmap of Pearson correlation coefficient for 2000 most variable expressed for WT organoid cells, eGBO‐derived tumors, and GBM patient samples. E) Compact representation of CNVs present in each subclone identified in eGBO‐derived tumors. F) 2D representation of malignant cell states. Each quadrant corresponds to a cellular state and the position of each dot reflects the relative meta‐modules scores for individual malignant cells. OPC‐like = oligodendrocyte‐progenitor‐like; NPC‐like = neural‐progenitor‐like; MES‐like = mesenchymal‐like; AC‐like = astrocyte‐like. G) Violin plots indicating module scores for recurrent cancer cell states in malignant cells from eGBO‐ and patient‐derived tumors. H) METAFlux nutrient profiles for metabolites commonly altered in GBM. Dot size represents the mean absolute cubic root normalized flux scores, and color represents the direction of flux.

Cancer cells are difficult to classify as distinct cell types,^[^ ^50^ ^]^ therefore we integrated our scRNAseq data with previously published data from 29 patient GBM samples^[^ ^51^ ^]^ to characterize eGBO‐derived tumor cells (**Figure** **5**A; Figure S7A, Supporting Information). Non‐malignant cells were identified based on previously reported marker genes^[^ ^51^ ^]^ corresponding to immune cells and oligodendrocytes (Figure 5B; Table S6, Supporting Information). Quantification of the proportion of each sample across malignant and non‐malignant cell types revealed that the non‐malignant cell types consisted primarily of non‐malignant patient cells and cells recovered from transplanted WT organoids while the majority cells recovered from transplanted eGBOs were classified as malignant (Figure 5C). Pearson correlation analysis also demonstrated that the transcriptional profiles of eGBO‐derived tumor cells were more similar to malignant patient cells, while recovered WT organoid cells were more similar to non‐malignant patient cells (Figure 5D).

Copy‐number variations (CNV) are a hallmark of malignant glioma cells, with sixteen broad events identified in GBM samples.^[^ ^52^ ^]^ We assessed the landscape of CNVs in eGBO‐derived tumors and patient‐derived GBM samples using a variational algorithm to characterize the copy number profile of clonal substructures within tumors from scRNAseq data^[^ ^53^ ^]^ (Figure 5D,E; Figure S7B, Supporting Information). Notably, we observed subclones within the PRO eGBO‐derived tumor cells with amplification in chromosome 7, while a deletion on chromosome 10 was observed in one of the MES eGBO‐derived tumor cell subclones. These observations are consistent with prior assessments in patient GBM samples,^[^ ^51^, ^52^ ^]^ which found that alterations in chromosomes 7 and 10 were most common despite high inter‐tumoral variability between patients and intra‐tumoral variability within subclones.

Prior characterization of inter‐tumoral and intra‐tumoral heterogeneity in patient GBM samples identified four recurrent cell states that occur across GBM cells.^[^ ^51^ ^]^ Consistent with these prior findings, we observe not only a heterogeneous distribution of cell states across patient‐derived tumor cells, but also mutation‐specific distributions of cell states in eGBO‐derived tumor cells (Figure 5F). MES eGBO‐derived tumor cells existed predominantly in the AC‐like state, with some cells falling in the OPC‐like or MES‐like states. PRO eGBO‐derived tumor cells were largely in the NPC‐like state, with some cells associated with the MES‐like or AC‐like states.

In addition to these GBM‐specific cell states, recurrent cell states that constitute general features of cancer have been identified (Table S6, Supporting Information).^[^ ^50a^ ^]^ Analyzing the module score of these recurrent cell states, we found that eGBO‐derived tumors recapitulate some aspects of patient‐derived GBM tumors (Figure 5G), including a subset of cells expressing cell cycle genes (Cycle module) and oxidative phosphorylation genes (Oxphos module). Interestingly, we also observe subtype‐specific enrichment of modules, such as the epithelial‐mesenchymal transition module (EMT) in MES eGBO‐derived tumor cells. These data indicate tumors formed by eGBOs recapitulate the heterogeneous composition of cell states found in human GBM.

Another critical characteristic of gliomas is altered cellular metabolism, specifically in the uptake of nutrients to fuel metabolic activity.^[^ ^54^ ^]^ Therefore, we performed metabolic flux balance analysis using a computational framework to infer the metabolic flux of various metabolites from scRNAseq data.^[^ ^55^ ^]^ Similar to patient‐derived tumors, both PRO and MES eGBO‐derived tumors exhibit consumption of glucose and glutamine along with secretion of lactate, suggesting utilization of aerobic glycolysis to produce energy (“the Warburg effect”).^[^ ^56^ ^]^ However, the inferred metabolic flux analysis revealed that the MES eGBO‐derived tumors, which appeared more invasive histologically, also exhibited increased consumption of acetates, which is a bioenergetic substrate for GBM and brain metastases.^[^ ^57^ ^]^

### 2.6 Developmental States in eGBOs Model Cancer Cell States Found During GBM Progression

**Figure 6:**
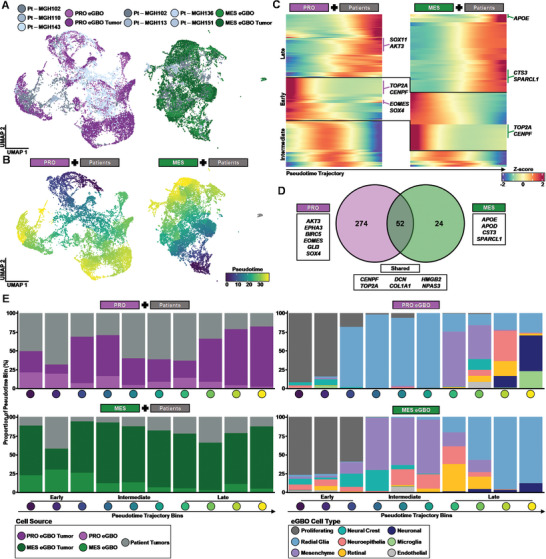
Trajectory analysis reveals developmental states govern GBM progression. A) UMAPs of integrated datasets for eGBOs, eGBO‐derived tumors, and GBM patient samples. The PRO dataset consists of PRO eGBOs, PRO eGBO‐derived tumor cells, and cells derived from GBM patients with a TP53 mutation. The MES dataset consists of MES eGBOs, MES eGBO‐derived tumor cells, and cells derived from GBM patients with a PTEN mutation. B) UMAPs showing the pseudotime trajectory of cells from a proliferating population to different cancer cell states for PRO and MES integrated datasets. C) Trajectory heat maps showing changes of gene expression across pseudotime for PRO and MES integrated datasets. D) Venn diagram of pseudotemporally autocorrelated genes from PRO and MES integrated datasets. E) Stacked bar plots showing the proportion of cells from eGBOs, eGBO‐derived tumors, and GBM patient samples (left) and the proportion of eGBO cell types (right) across pseudotime bins.

Recently, it has been proposed that cancer cell states in GBM and other cancers are constrained within the developmental hierarchy of the cell that originates tumor formation.^[^ ^10^, ^11^, ^58^ ^]^ Our eGBOs combined with patient tumor samples offer a unique model to investigate this hypothesis. Therefore, we generated two integrated datasets consisting of eGBOs, eGBO‐derived tumors, and malignant patient‐derived GBM samples with comparable mutational profiles (**Figure** **6**A; Figure S7C, Supporting Information).

We performed single‐cell trajectory analysis to characterize the changes in gene expression as proliferative cells transition to malignant cell states (Figure 6B). Clustering of genes that have expression correlated to pseudotime trajectory revealed three distinct transcriptional stages (Figure 6C). Interestingly, we found a larger number of genes correlated with pseudotemporal stages in the PRO integrated dataset compared to the MES integrated dataset (Figure 6D). Shared pseudotemporally correlated genes between both integrated datasets included genes associated with proliferation (*CENPF* and *TOP2A*), cancer‐associated fibroblasts^[^ ^59^ ^]^ (*DCN* and *COL1A1*) and neurodevelopment^[^ ^60^ ^]^ (*HMGB2* and *NPAS3*). Pseudotemporally correlated genes specific to MES integrated dataset included differentiation markers found in astrocytes or oligo‐lineage cells, such as *APOE* and *APOD*, that also mediate pro‐inflammation and proliferation signatures in GBM.^[^ ^11^, ^61^ ^]^ Genes associated with GBM tumor‐initiating cells^[^ ^62^ ^]^ (*AKT3, EPHA3, BIRC5*, and *EOMES*) and radial glia cells (*GLI3* and *SOX4*) were unique to the pseudotemporally correlated gene list of the PRO integrated dataset. To investigate changes in cell states across tumor progression, we binned cells based on their pseudotime ordering and quantified the proportion of cell types in each bin (Figure 6E). The distribution of cell types across the pseudotime further delineated similarities and differences between the PRO and MES integrated datasets. In both datasets, eGBO cells along with patient‐ and eGBO‐derived tumor cells were well distributed across the pseudotime ordering. Additionally, the early pseudotime stages in both datasets consisted of primarily of proliferating cells from eGBOs prior to transplantation. Notably, the cell populations in the intermediate and late pseudotime stages appear to be swapped in between the two datasets. Specifically, radial glial cells from eGBOs appear in the intermediate pseudotime stage in the PRO dataset, while mesenchymal and other cell types are present in the late pseudotime stage. In the MES dataset, on the other hand, mesenchymal and other cell types appear in the intermediate pseudotime stage, while radial glial cells from eGBOs are primarily found in the late pseudotime stage. This is consistent with previous observations in human GBM samples demonstrating that proliferating GBM stem cells exist on an axis of PRO‐MES, which contributes to the heterogeneity observed in GBM.^[^ ^63^ ^]^ Additionally, the presence of cells from eGBOs and both patient‐ and eGBO‐derived tumors in the late pseudotime stage of suggests that early developmental states observed in eGBOs are representative of malignant cell states that arise during GBM progression. Together, these data highlight that subtype‐specific eGBOs can model different developmental states that contribute to the heterogeneity of GBM during tumor progression.

## 3 Discussion

The lack of effective treatments for GBM highlights the need for improved preclinical models that replicate human tumorigenesis and progression. By introducing GBM subtype‐specific mutations using genetic engineering methods, we have developed eGBOs as an in vitro model system for GBM. The combination of brain organoids and genetically engineered oncogenic aberrations enabled us to characterize the disruption of developmental pathways and the progression of cancer cell states that are characteristic not only of GBM, but also a broad range of cancers. Further, GBM subtype‐specific mutations within eGBOs enabled characterization of the progression of different GBM subtypes through developmental states.

Prior genetically engineered organoid models of brain tumors have exhibited many features of cancer, including malignant cellular identities and the capacity for in vivo expansion and invasion.^[^ ^7^, ^12^ ^]^ Induced expression of the oncogenic HRas^G12V^ mutation, which is not common in patients, was demonstrated as a proof of principle that cerebral organoids could be used as a model for tumor formation.^[^ ^12a^ ^]^ Similarly, inducing specific combinations of oncogene expression or mutations via nucleofection promoted GBM‐like overgrowths within developing organoids that could be utilized for drug screening.^[^ ^12b^ ^]^ Building on this work, we targeted the *TERT* promoter region to create a defined genetic background that recapitulates one of the earliest GBM mutations that provides a selective advantage for malignant cells.^[^ ^8a^ ^]^ We further generated subtype‐specific eGBOs by targeting some of the common mutations found in subsets of patient samples. Orthotopic transplantation of eGBO cells resulted in tumors that exhibited many characteristics of GBM, including histological presentation, spatial organization, and cell state heterogeneity. We observed distinct differences in spatial architecture and cell states in tumors formed by PRO and MES eGBOs that can be attributed to the differences in their respective genetic backgrounds. Notably, these differences correspond to characteristics of human GBM samples that contribute to inter‐ and intra‐tumor heterogeneity.

While eGBOs are a promising model for further elucidating human brain tumor biology and screening tool for potential therapeutic approaches, they still lack components that are critical for tumor progression. For example, existing vasculature is involved in multiple GBM processes, such as glomeruloid body formation^[^ ^64^ ^]^ and pseudopalisading necrosis.^[^ ^65^ ^]^ Interestingly, a small population of endothelial cells was present in eGBOs suggesting that they may be suitable model to study angiogenic signaling during GBM progression. Integration of eGBOs and vascularized organ‐on‐chip models can also enable investigation of early tumor interactions with existing vascular networks and tumor invasion. Additionally, many genes are mutated in GBM, and the mutational burden can vary significantly not only between patients, but also within patient samples. Tumor mutational burden may provide a useful biomarker for predicting immunotherapy success in GBM.^[^ ^66^ ^]^ However, we did not explore survival following orthotopic implantation of eGBOs and this is an interesting avenue for future studies. Extended characterization of in vivo tumor formation from eGBOs would also enable better assessment of how well these tumors recapitulate the mutational landscape observed in advanced patient tumors. While we only explored a small subset of GBM‐associated mutations, our results demonstrate that eGBOs can be utilized to interrogate how specific mutation combinations drive different features of GBM.

## 4 Conclusion

Our single‐cell and spatial transcriptomic analyses of eGBOs provides an important validation of engineered cancer organoid models and demonstrate their utility as a human‐relevant model of GBM tumorigenesis for future preclinical drug development. Specifically, we show that eGBOs can model different aspects of GBM progression based on specific combinations of mutations and future development can focus on investigating the pathogenesis of different, patient‐specific mutations. This model offers a complementary approach to existing and emerging methods for investigating tumor biology and pathophysiology. For example, combining eGBOs and single‐cell proteomics, using methods such CITE‐seq and single cell mass spectrometry, may elucidate how the proteome specifically regulates the development of GBM. Ultimately, eGBOs have the potential to enable early drug discovery of therapeutics designed to target patient‐specific genetic backgrounds.

## 5 Experimental Section

### Ethics Statement

Human stem cell research related to this study has been approved by the Washington University School of Medicine Institutional Review Board and Embryonic Stem Cell Research Oversight Committee (IRB ID: 201709124 and ESCRO# 17‐005). The Washington University School of Medicine Institutional Animal Care and Use Committee (IACUC) approved the protocols for experiments involving animals (#21‐0083). Mice were housed in the Washington University Department of Comparative Medicine animal facilities according to standard protocols.

### Culture and Genetic Engineering of hPSCs

H1 hPSCs (CVCL_9771) were maintained on Matrigel‐coated T75 culture flasks in mTeSR1 media (Stemcell Technologies; 85850). During passaging, media was supplemented with 10 µM Y‐27632 (Abcam; ab120129) to inhibit Rho kinase‐mediated apoptosis. Genetically engineered H1 hPSCs cell lines were generated by the Genome Engineering & Stem Cell Center at Washington University School of Medicine in St. Louis. Briefly, H1 hPSCs were transfected with Cas9 recombinant protein and synthetic single guide RNA to introduce a C > T mutation within the telomerase reverse transcriptase (*TERT*) gene promoter.^[^ ^13a^ ^]^ The H1 TERT mutant cell line was then further to modified, by the same procedure, to introduce either a point mutation targeting the *TP53* gene or knockouts mutations in the *PTEN* and *NF1* genes. CRISPR guide RNA sequences are listed in Table S7 (Supporting Information).

### Immunocytochemistry

For immunocytochemistry (ICC), cells were fixed in 4% paraformaldehyde (Electron Microscopy Science; 15714) for 30 min at room temperature (RT). Fixed cells were then incubated at RT in ICC solution consisting of 0.1% Triton X (Acros Organics; 327371000) and 5% donkey serum (Jackson Immunoresearch; 01700‐121) in phosphate buffered saline (Fisher; MT21040CV), for 30 min to permeabilize cell membranes and block non‐specific binding sites. Samples were subsequently treated with NANOG primary (R&D Systems; AF1997, Lot #KKJ0821031; 1:300 dilution) and Alexa Fluor 488 secondary (Invitrogen; A11055, Lot #2300215) antibodies in ICC solution overnight at 4 °C and 2 h at RT, respectively. DAPI (Invitrogen; D1306, Lot #1583092; 1:10000 dilution) was used for nuclear staining. Samples were incubated in DAPI for ≈12 min at RT, washed with ICC solution and stored in PBS until imaging.

### Quantitative PCR Analysis

RNA was extracted from cells using the RNeasy Mini Kit (Qiagen; Cat #74104) following the manufacturer's instructions. Quantification and quality assessment of RNA was performed using a BioTek Synergy H1multimode microplate reader. Complementary DNA (cDNA) was synthesized from extracted RNA using the using the High‐Capacity cDNA Reverse Transcription Kit (Applied Biosystems; 129382310MG) and a T100 thermocycler (Bio‐Rad). The TaqMan Universal Master Mix (Applied Biosystems; 4440040) and predesigned TaqMan Gene Expression Assays (Applied Biosystems; 4331182) were used to detect mRNA transcript levels on a QuantStudio 6 Pro Real‐Time PCR System (Applied Biosystems; A43180). Results were analyzed using the ΔΔCt methodology with GAPDH used as a housekeeping gene. Primers for qPCR are listed in Table S8 (Supporting Information).

### Brain Organoid Differentiation

Brain organoids were generated from H1 hPSC lines following a previously described protocol,^[^ ^19^ ^]^ with minor modifications. Briefly, H1 hPSCs were cultured in hPSC Media on Matrigel‐coated T75 flasks. On day 0 of differentiation, cells were transferred into low‐attachment 96‐well plates at ≈10000 cells/well to generate embryoid bodies. On day 2, each embryoid body was transferred to a single well of a 24‐well plate with Neural Induction Media. On day 6, developing neural organoids were embedded in Matrigel droplets and transferred to a petri dish with Cerebral Organoid Differentiation Media. On day 10, petri dishes were transferred to an orbital shaker at 100 rpm and fed every 4 days with Organoid Differentiation Media. Media formulations and additional details are provided in Table S1 (Supporting Information).

### Organoid Morphology Analysis

The Fiji distribution of ImageJ was used to perform area and circularity measurements.^[^ ^67^ ^]^ Briefly, bright‐field images were converted to 8‐bit greyscale, then binarized to delineate the borders of the developing organoid. Edges of the organoid area was defined using the wand tracing tool. Area and shape descriptors were measured using the default Measure functions. Circularity was calculated as 4π(area/perimeter^2^), with a value of 1.0 indicating a perfect circle.

### Orthotopic Xenotransplantation

Dissociated brain organoid cells were injected into the brain of seven‐week‐old female athymic nude mice as previously described.^[^ ^12a^ ^]^ Briefly, ≈260000 cells were injected stereotactically into seven‐week‐old female athymic nude mice (n = 5 per condition). Mice were anesthetized with isoflurane prior to all major procedures including stereotactic tumor implantation, MRI acquisition, and euthanasia. For stereotactic injection, animals received analgesia via buprenorphine‐SR injection lasting ≈72 h post‐operatively. Eye gel was used throughout the procedure and mice were kept on a warming pad. Post‐operatively, animals recovered in a warmed environment until fully awake, then transferred to an isolated animal room for up to 24 h without disturbances. Injections targeted the right putamen at coordinates 1 mm rostral to bregma, 2 mm lateral, and 2.5 mm deep. To track tumor growth, MRI was performed on a Bruker BioSpec 9.4T MRI (Bruker, Billerica, MA) using an 86 mm inner diameter volume transmitter coil and a 4‐channel mouse brain CryoProbe array receiver coil. Prior to imaging, mice were anesthetized with 1‐1.5% isoflurane on a circulating warm water pad to maintain body temperature. Fat‐suppressed T2‐weighted RARE images were acquired of the brain using the following parameters: TR = 2500s, TE = 33s, matrix size = 256 × 256, spatial resolution = 700 µm, 9 slices at 5 mm thickness, RARE factor = 9, 1 average, acquisition time = 1 min. Image analysis was done using the semi‐automatic segmentation analysis software ClinicalVolumes (ClinicalVolumes, London, UK). Mice euthanized to harvest brain tissue for histopathological analysis were subjected to isoflurane anesthesia. Thereafter, the thorax was opened using a scalpel and small scissors to expose the pulsating heart. Using a small gauge needle, the left ventricle was punctured and perfused with ice cold phosphate−buffered saline to remove blood followed by approximately ice cold 4% paraformaldehyde by perfusion pump or by gravity over 10–15 min. Mice were then decapitated and the brain isolated for histologic analysis.

### Tumor Dissociation and Histology

Three months after transplantation, mice were sacrificed, and their brains were harvested. For scRNAseq analysis, tumors were dissociated using the Brain Tumor Dissociation Kit (Miltenyi Biotec, 130‐095‐942). Myelin was removed using Myelin Removal Beads II (Miltenyi Biotec, 130‐096‐731). Mouse cells were removed using the Mouse Cell Depletion Kit (Miltenyi Biotec, 130‐104‐694). Dissociated cells from two mice per condition were pooled together. The other three brains were processed for histology. Briefly, samples were fixed with 4% PFA cryopreserved with 30% sucrose, and flash frozen. Fixed samples were sectioned at 10 µm thickness along the coronal plane using the tissue near the needle tract for cell injection based on stereotactic coordinates. Brain sections were stained with hematoxylin to mark the cell nuclei and eosin to stain the extracellular matrix and cytoplasm. Additional sections were stained for GFAP (Sigma‐Aldrich; IF03L, Lot #D00109967, Z0334; 1:1000 dilution) and VIM (Abcam, ab8978, Lot # GR320068‐5; 1:20 dilution).

### Single‐Cell RNA Sequencing

Cell suspensions were delivered to the Washington University in St. Louis Genome Technology Access Center (GTAC) for library preparation and sequencing. For library preparations, the Chromium Next GEM Single Cell 3ʹ Reagent Kit v3.1 (10x Genomics) was used following the provided protocol. The library was sequenced with a NovaSeq 6000 System (Illumina) at the recommended 26×98 bp. To label cells with unique hashtags, approximately ten organoids per condition were pooled and dissociated in TrypLE for 15 min with gentle agitation every 5 min. Single cell solutions were resuspended in 200 µL of cell staining buffer (BioLegend, 420201) plus 1 µg of TotalSeq‐A hashtag antibodies (BioLegend, 399907) and incubated for 30 min on ice. After three washes in 1 mL of cell staining buffer, cells were resuspended in DMEM at a concentration of 1000cells/µL and pooled together.

### In Situ Sequencing

ISS was performed at GTAC using Xenium platform (10x Genomics), following protocols provided by the company. Sections were prepared according to the “Xenium In Situ for FFPE‐Tissue Preparation Guide” (CG000578 Rev C, 10X Genomics) protocol. Briefly, 5 µm sections were cut and carefully placed onto the Sample Area of a Xenium slide (PN‐1000465). The slides were then placed in a drying rack at room temperature for 30 min to remove excess water, followed by a 3 h incubation at 42 °C on a Xenium Thermocycler Adapter plate positioned in a thermocycler (BioRad). Subsequently, the slides were stored overnight at room temperature with a desiccant for further drying. The following day, Xenium slides were processed following the “Xenium In Situ for FFPE‐Deparaffinization and Decrosslinking” protocol (CG000580 Rev C, 10X Genomics). The Xenium slides were then assembled into Xenium cassettes (PN‐1000566, 10X Genomics), which allow for the incubation of slides on the Xenium Thermocycler Adapter plate in a PCR machine with a closed lid for precise temperature control. The slides were processed using the “Xenium Slides and Sample Prep Reagents” kit (PN‐1000460, 10X Genomics), beginning with an incubation in a decrosslinking and permeabilization solution at 80 °C for 30 min. The Xenium slides were then processed according to the “Xenium In Situ Gene Expression” user guide (CG000582 Rev D, 10X Genomics) for the remaining slide preparation steps. The slides were incubated at 50 °C for 17 h with the pre‐designed gene expression probe set, “Xenium Human Brain Gene Expression”. This was followed by a series of wash and enzymatic steps, including a 30 min post‐hybridization wash at 37 °C, a 2 h ligation at 37 °C, and a 2 h amplification step at 30 °C. After additional washes, the slides were treated with an autofluorescence quencher and a stained with DAPI. Processed Xenium slides, assembled in Xenium cassettes, were subsequently imaged using the Xenium Analyzer, following the guidelines provided in the “Xenium Analyzer User Guide: (CG000584 Rev B, 10X Genomics).” The two Xenium slides/cassettes were then loaded into the instrument, initiating the Analyzer's “sample scan” process. This scan produced images of the fluorescent nuclei in each section. Upon completion, these images were utilized to determine the regions within the scan area to be included in the instrument's comprehensive scan of the gene expression probe set.

### Single‐Cell RNA and In Situ Sequencing Data Analyses

Datasets were analyzed using a custom containerized Linux environment consisting of JupyterLab v4.0.11, R v4.4.1, and Python v3.10.13. Initial processing and quality control filtering of scRNAseq and ISS data was performed using Seurat v5.^[^ ^40^ ^]^ Unsupervised annotation of eGBO cell types was performed using SingleR^[^ ^68^ ^]^ and VoxHunt.^[^ ^32a^ ^]^ GSEA was performed using fgsea.^[^ ^69^ ^]^ Inferred GRN analysis was performed using SCENIC.^[^ ^31^ ^]^ Additional analysis of ISS data was performed using Scanpy,^[^ ^70^ ^]^ Squidpy,^[^ ^71^ ^]^ CellCharter,^[^ ^41^ ^]^ and scvi‐tools.^[^ ^72^ ^]^ Inferred CNV analysis was performed using SCEVAN.^[^ ^53^ ^]^ Inferred metabolic flux balance analysis was performed using METAFlux.^[^ ^55^ ^]^

### Statistical Methods

Statistical analyses were performed using GraphPad Prism 8.0.1 software (GraphPad Software, San Diego, CA). Statistical analyses used included parametric unpaired t‐test using the Holm‐Sidak method and two‐way ANOVA with matching factors corrected for multiple comparisons using Dunnett's multiple comparison test and the Geisser‐Greenhouse correction without sphericity. A confidence level of 95% was considered significant with designations of *P < 0.05, **P < 0.01, and ***P < 0.001 (two‐tailed). Results without statistical significance denoted can be assumed to be of no significant difference. All values were expressed as mean ± standard deviation.

## Conflict of Interest

M.I. has stock in Vertex Pharmaceuticals. A.H.K. is a consultant for Monteris Medical and has received research grants from Stryker for a clinical outcomes study about a dural substitute, which have no direct relation to this study. J.R.M. was an employee of and has stock in Sana Biotechnology. The remaining authors declare no competing interests.

## Author Contributions

M.I., A.H.K., and J.R.M. designed the project and experiments. M.I. performed in vitro experiments and performed the computational analyses. D.A., P.A.D., and X.Q. generated CRISPR‐edited stem cell lines. R.H.H., C.M., R.T.C., and T.W. performed in vivo experiments. M.I. and S.D. performed assessment of histological samples. M.I. wrote the manuscript. All authors revised and approved of the manuscript.

## Supporting information



Supporting Information

Supporting Tables

## Data Availability

The scRNAseq and spatial transcriptomics data generated in this study have been deposited in the Gene Expression Omnibus (GEO) database and are accessible via accession codes GSE283496 (scRNAseq) and GSE283497 (spatial transcriptomics).
