# Supplementary material for: Genetically Engineered Brain Organoids Recapitulate Spatial and Developmental States of Glioblastoma Progression
Source: Adv Sci (Weinh). 2025 Jan 21;12(10):2410110. doi: 10.1002/advs.202410110 (PMC11905097; doi:10.1002/advs.202410110)

Supporting Information

**Genetically Engineered Brain Organoids Recapitulate Spatial and Developmental States of Glioblastoma Progression**

*Matthew Ishahak, Rowland H. Han, Devi Annamalai, Timothy Woodiwiss, Colin McCornack, Ryan T. Cleary, Patrick A. DeSouza, Xuan Qu, Sonika Dahiya, Albert H. Kim*, and Jeffrey R. Millman**

**Figure S1.** Characterization of stem cells harboring GBM-associated mutations. A) Depiction of genetic mutations introduced into hPSCs. B) Brightfield (top, scale bars = 500µm) and immunofluorescence imaging (bottom, scale bars = 50µm) of hPSCs indicating normal stem cell morphology and expression of the pluripotency marker, NANOG. C) NGS results demonstrating successful gene editing in engineering hPSCs (n = 4).


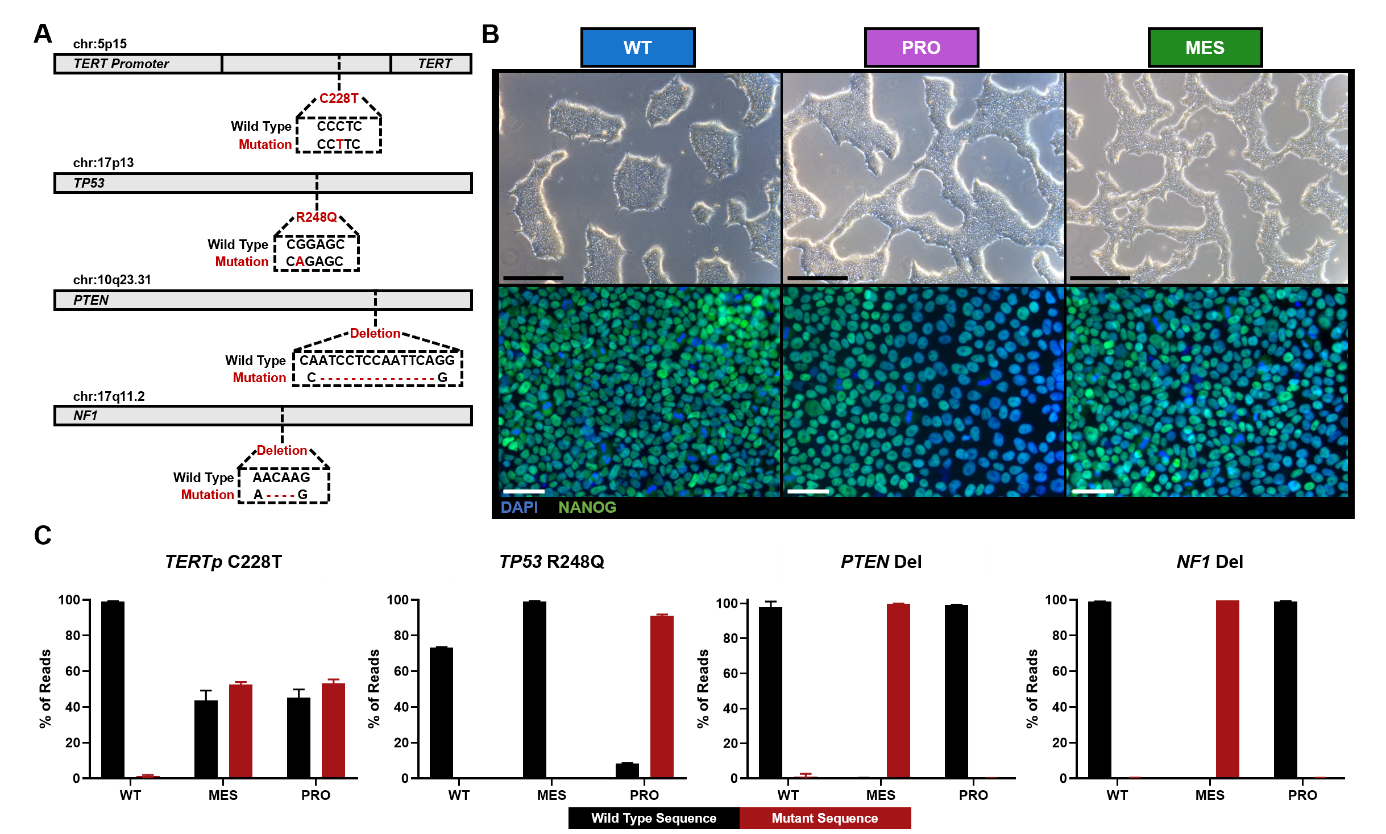


**Figure S2.** Hashtag demultiplexing and unsupervised cell type annotation in scRNAseq data. A) Schematic of multiplexed scRNAseq workflow. Briefly, organoids are dissociated into single cells and labeled with a unique hashtag oligonucleotide (HTO). Samples are then pooled and scRNAseq is performed. Finally, samples are demultiplex computationally. Created using BioRender.com icons. B) Heatmap showing expression of HTOs correctly defines each condition. C) Heatmap of genes targeted by CRISPR editing to mimic GBM subtypes. D) Heatmap of similarity index calculated by VoxHunt demonstrating Louvain clusters most strongly resemble regions of the developing pallium, which corresponds to the cerebral cortex. E) UMAP with individual cells annotated based on correlation to cell types from a published brain organoid atlas using SingleR. F) Heatmap of correlation scores for individual cells calculated using SingleR.


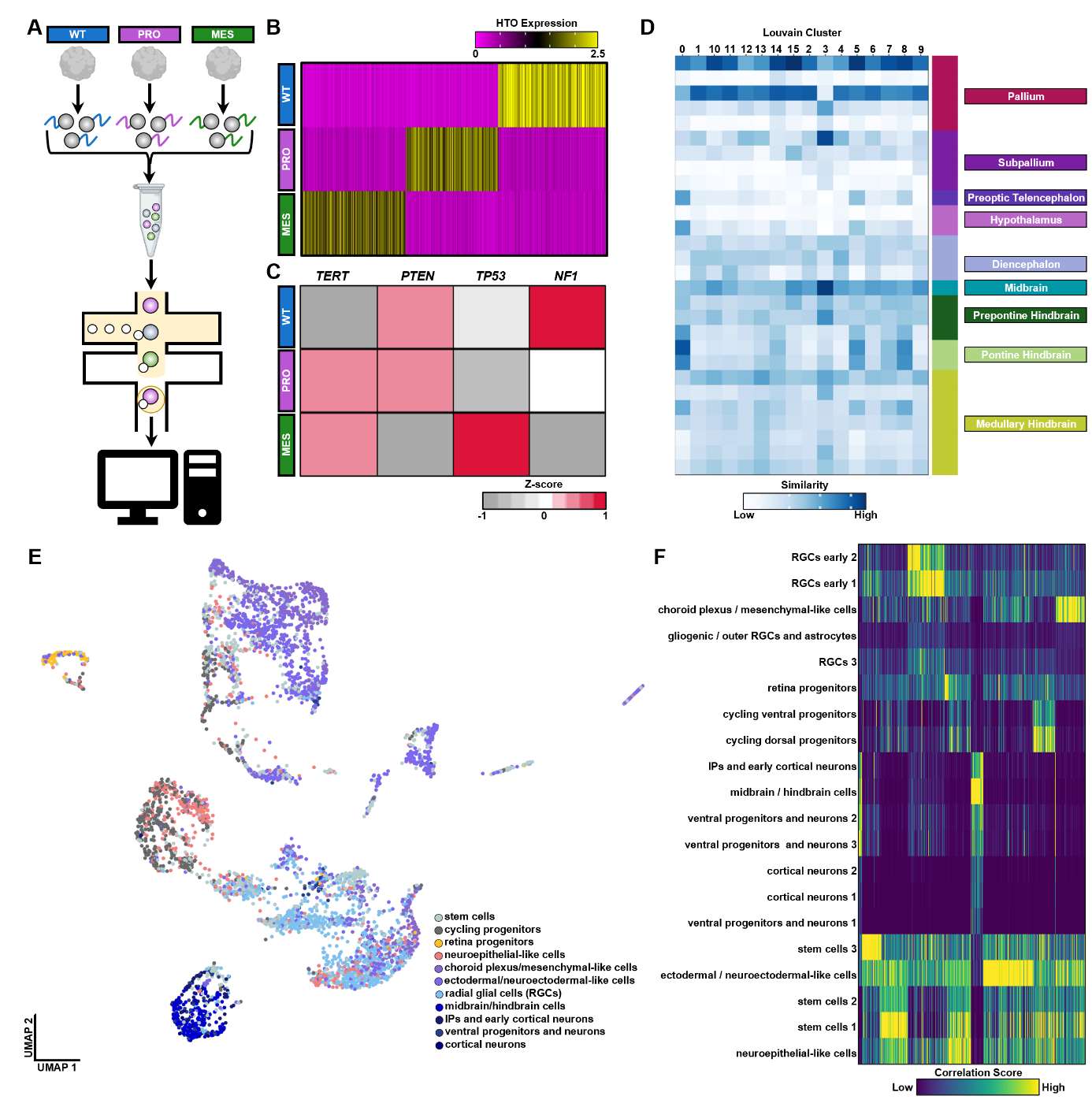


**Figure S3**. Analysis of iGRNs reveals activation of oncogenic gene regulatory networks in eGBOs. A) UMAP of WT, PRO, and MES organoids clustered by iGRN activity. B) Rank order plot of iGRN regulons sorted by regulon specificity score (RSS). C) Scatter plot of iGRN regulons. Red dots represent the top 50% of variable regulons based on standardized variance. D) Feature plots indicating iGRN activity of differentially active regulons (top) and gene expression of the corresponding TF (bottom).


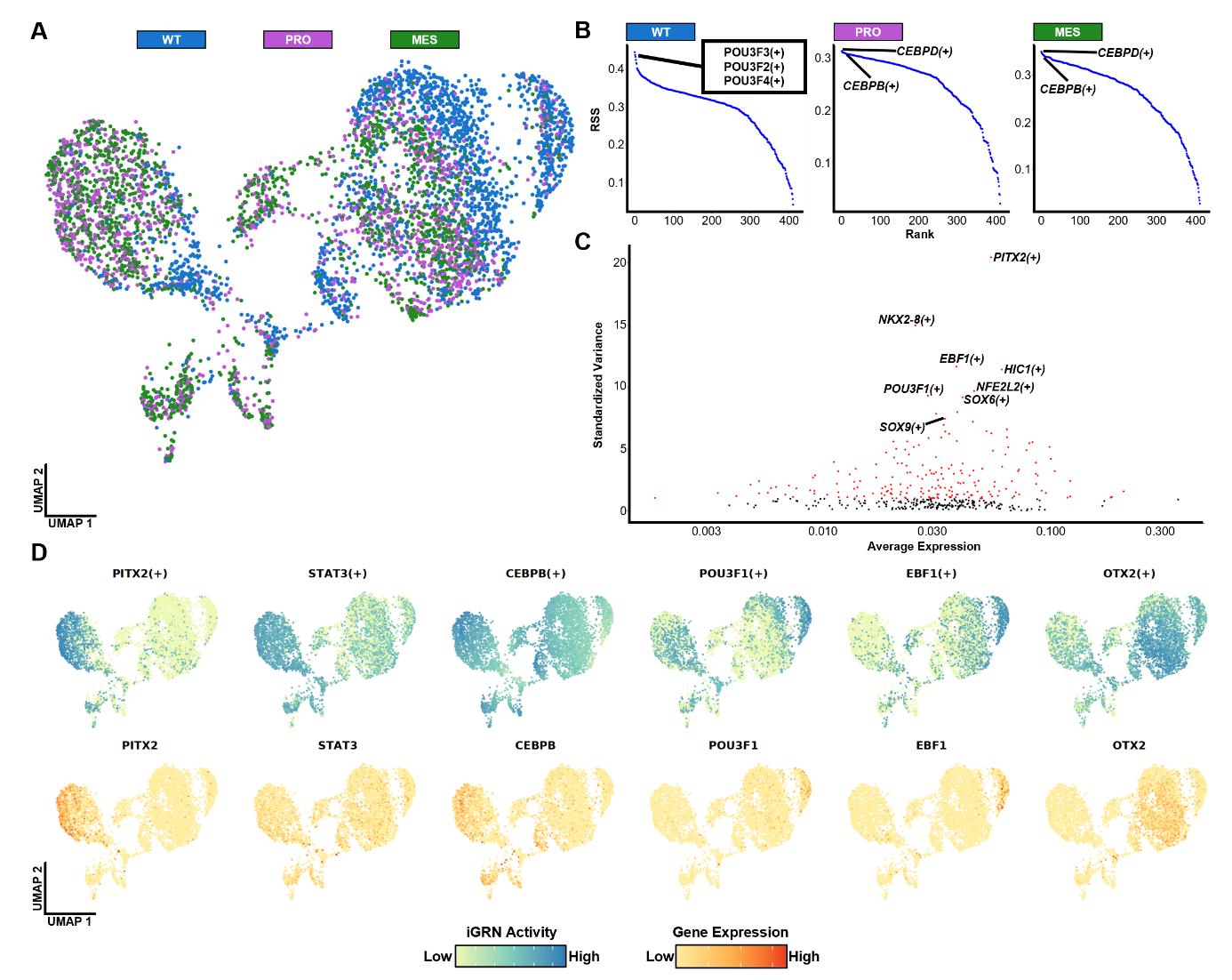


**Figure S4.** Analysis of spatially resolved transcriptomics in eGBOs. A) Spatial gene expression of genes in WT organoids and eGBOs. B) Cluster stability (y axis) for a range of number of clusters (x axis) for WT organoid and eGBO spatial sequencing samples. C) Cell type neighborhood enrichment heatmaps for WT organoids and eGBOs.


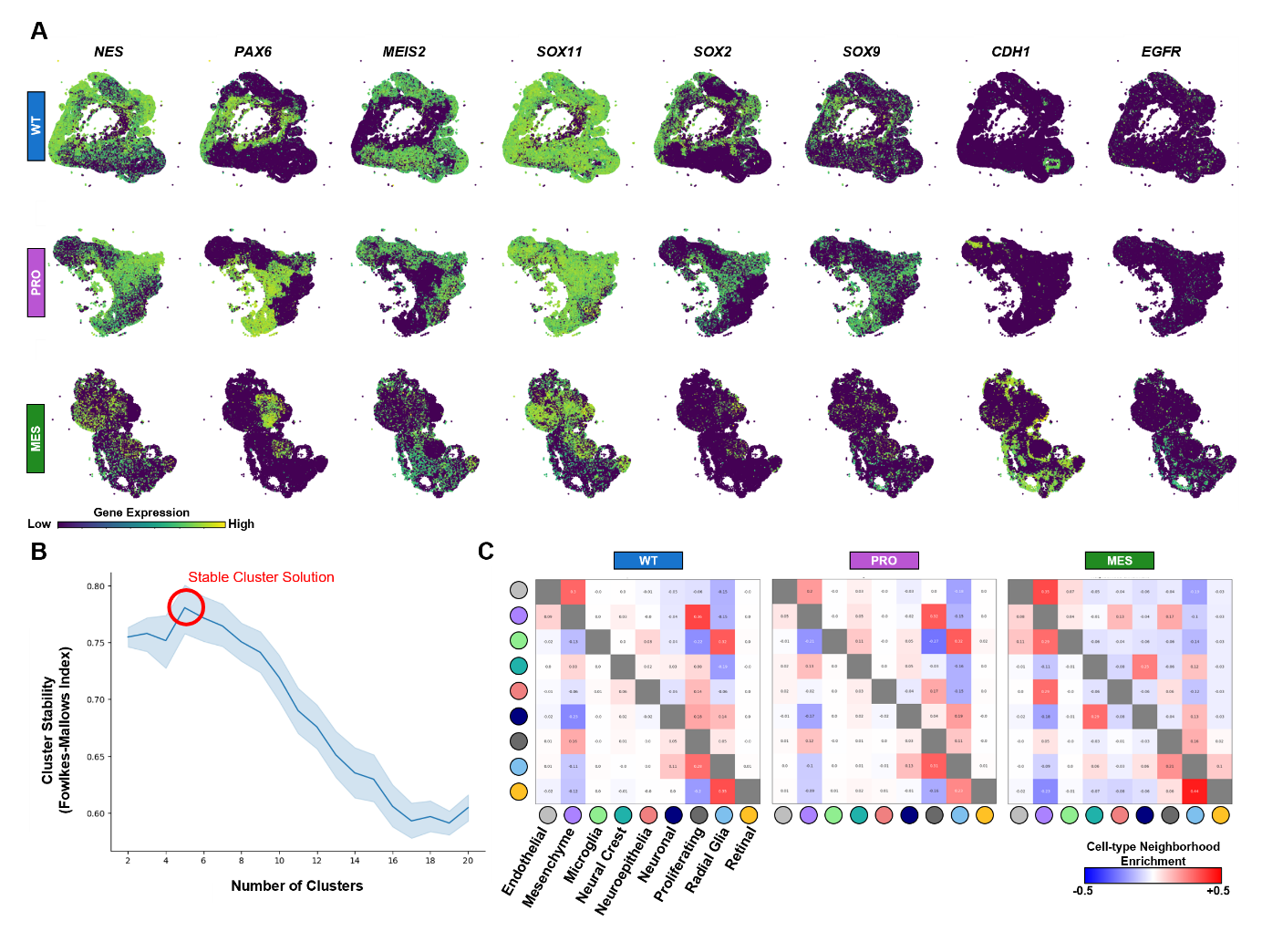


**Figure S5.** Characterization of tumor growth following orthotopic implantation of eGBOs. A) Representative T2-post contrast MRIs of mouse brains from 4-weeks to 12-weeks post implantation. B) Survival graph indicating the proportion of the orthotopic implantation cohorts surviving without presenting tumor-growth. C) Tumor size quantification based on area of contrast-enhancing lesions observed in MRIs at 8-weeks and 12-weeks post implantation (n=5). D) Histological assessment of GFAP (left) and VIM (right) in brain sections of mice that received cells from either PRO or MES eGBOs (Scale bars = 500µm). Insets provide zoomed in view on malignant regions highlighting differences between PRO and MES eGBO-derived tumors (Scale bars = 100µm).


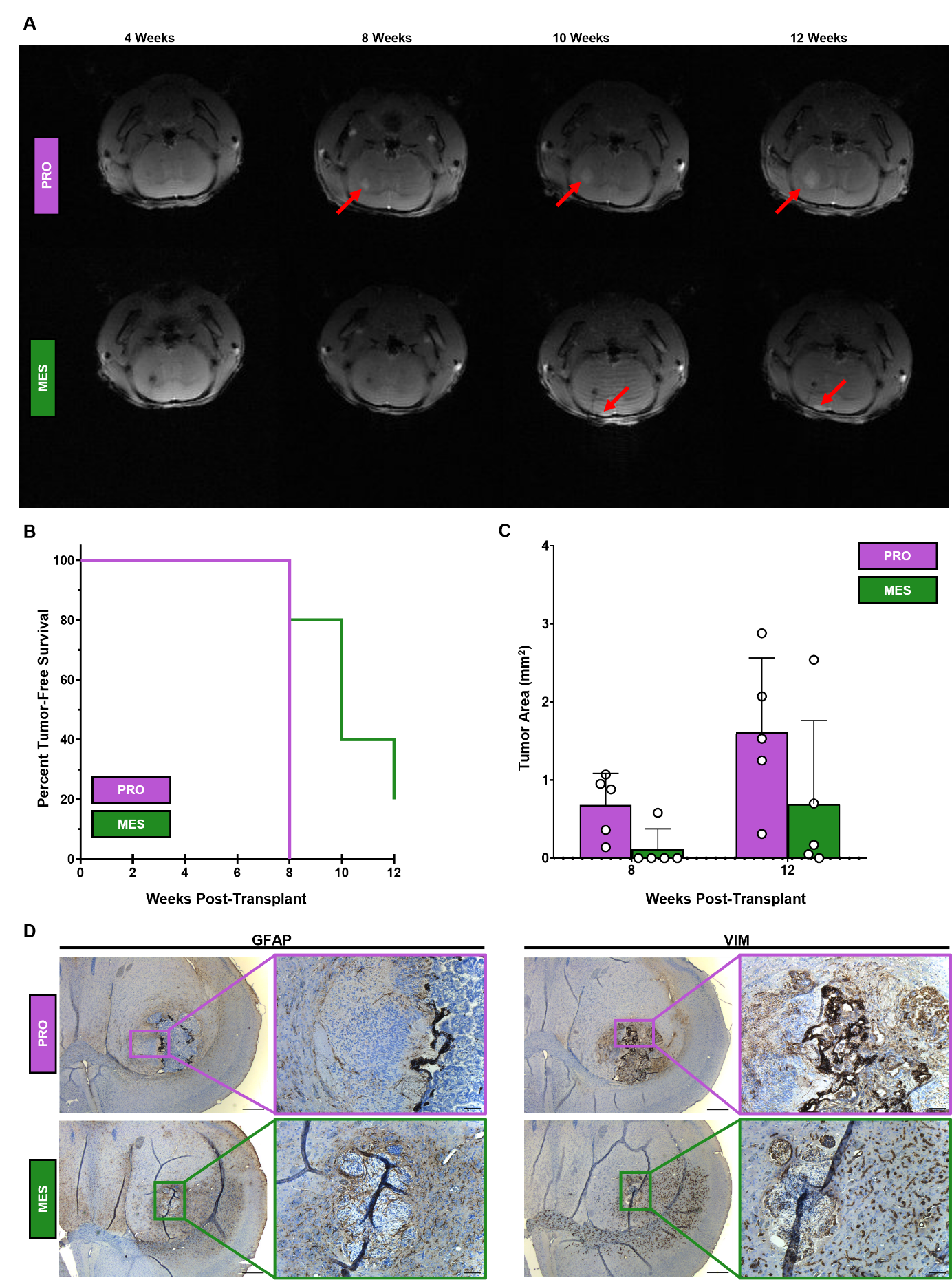


**Figure S6.** Spatially resolved transcriptomics analysis of tumors formed by eGBOs. A) H&E images (top row) predicted distribution of transcriptional subtypes (middle row) and predicted aggressive scores (bottom row) for a sample tumor from the TCGA cohort and tumors generated by eGBOs. B) Spatial gene expression profile of tumor-associated genes from the 10X Xenium brain panel. C) Venn diagram of spatially autocorrelated genes in brain sections from WT organoid and eGBO spatial sequencing samples. D) Cluster stability (y axis) for a range of number of clusters (x axis) for brain sections from WT organoid and eGBO spatial sequencing samples. E) Spatial gene expression of marker genes for different brain regions in spatial sequencing samples from WT organoid and eGBOs.


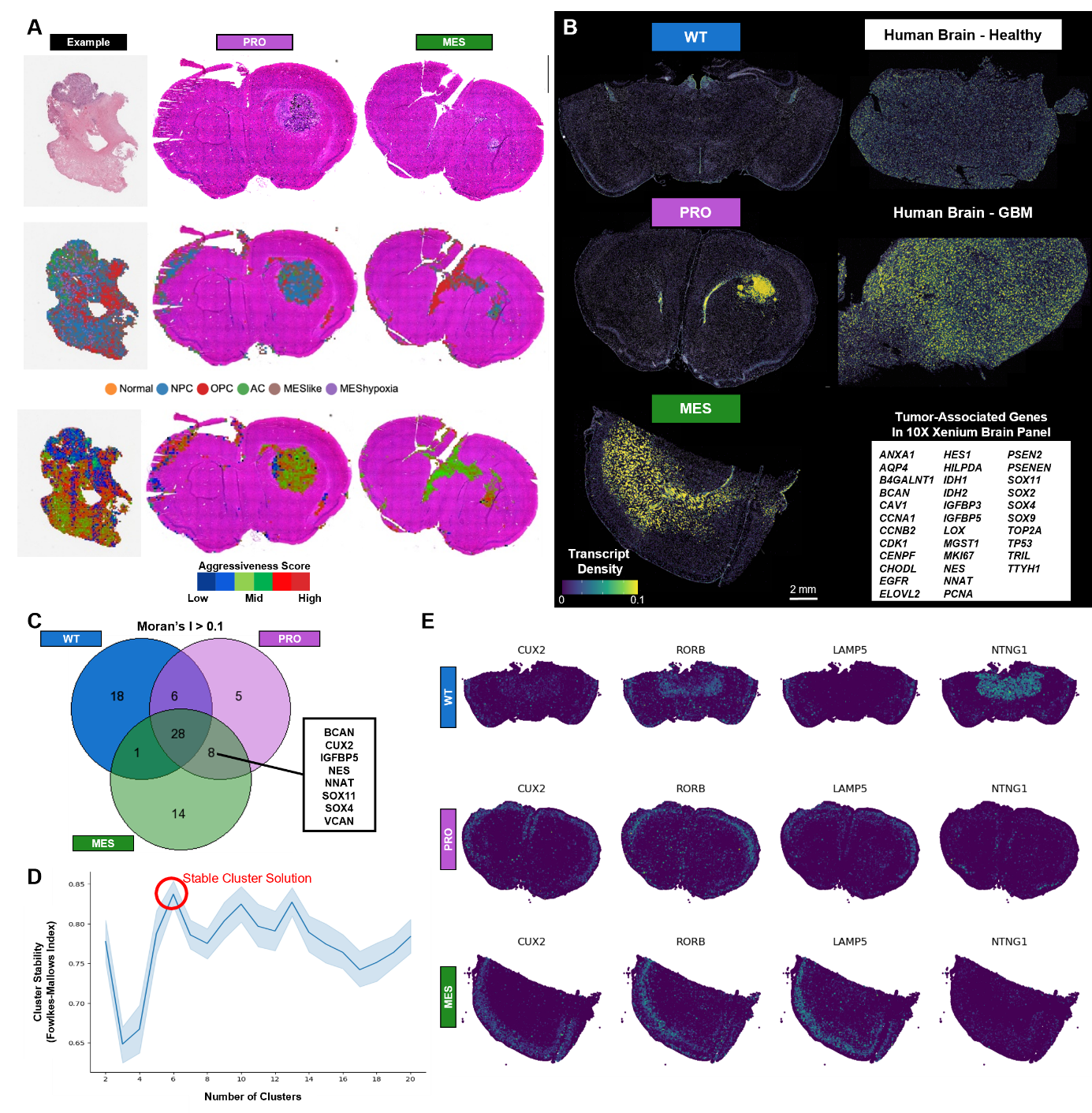


**Figure S7.** Inferred copy number aberrations in patient- and eGBO-derived tumors. A) UMAP of cells recovered from 29 GBM patient samples (21 adult and 8 pediatric). B) Compact representation of CNVs present in each subclone identified in a subset of patient GBM samples with similar mutations to eGBOs. C) UMAPs of integrated eGBO, eGBO-derived tumor, and patient-derived tumor cells indicating the distribution of eGBO cell types intermixed with tumor-derived cells.


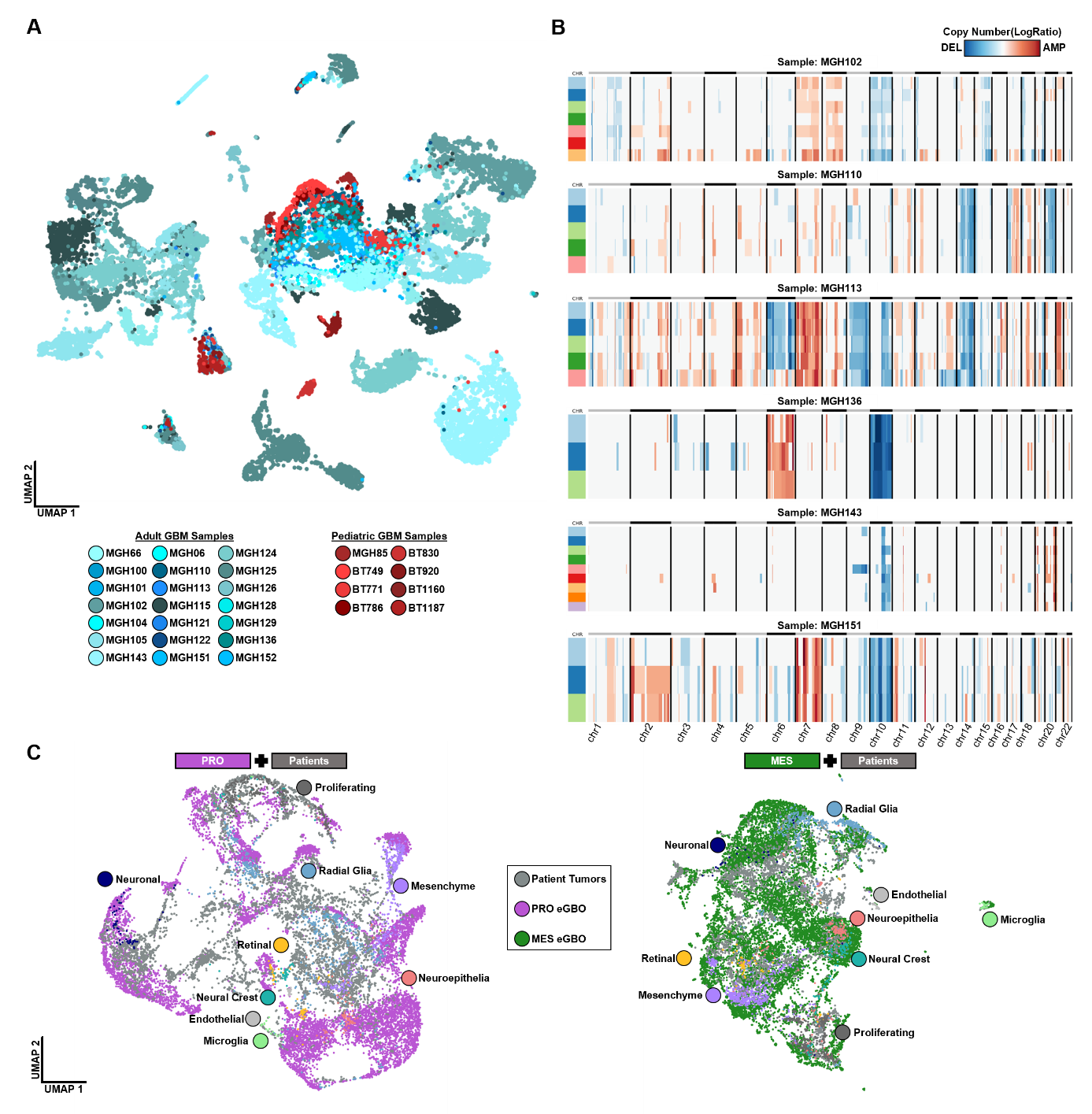

Supplement: Supplementary file 1 — Supporting Information [file ADVS-12-2410110-s002.docx]
